# Neuroimaging findings in hospitalized patients with COVID-19

**DOI:** 10.1186/s43055-022-00698-z

**Published:** 2022-01-10

**Authors:** Ahmed Abdelzaher, Mohammad AlQatam, Lamyaa Alsarraf, Mohamed H. Beheiri, Shehata Farag Shehata, Nermeen A. Elsebaie

**Affiliations:** 1 Diagnostic and Interventional Radiology Department, Alexandria Faculty of Medicine, Alexandria University, Shamplion street, Alexandria, 21131 Egypt; 2 Medical Imaging Department, Mubarak Al-Kabeer Hospital, Ministry of Health, Kuwait City, Kuwait; 3 Medical Imaging Department, Ibn Sina Hospital, Ministry of Health, Kuwait City, Kuwait; 4 Medical Statistics and Biomedical Informatics Department, High Institute of Public Health, Alexandria University, Alexandria, Egypt

**Keywords:** Corona virus disease 19, Ischemic infarcts, Venous sinus thrombosis, Posterior reversible encephalopathy syndrome

## Abstract

**Background:**

Variable neuroimaging findings have been reported in patients with coronavirus disease 2019 (COVID-19). In addition to respiratory symptoms, many neurologic manifestations of COVID-19 are increasingly reported and variable neuroimaging findings have been observed in patients with COVID-19. Our aim was to describe findings observed in hospitalized patients with COVID-19, presenting with acute neurologic manifestations and undergoing computed tomography (CT) or magnetic resonance imaging (MRI) of the brain.

**Methods:**

We performed a retrospective study involving patients with laboratory-confirmed SARS-COV-2 infection, admitted to our hospital between July 1 and December 30, 2020. Patients who presented with acute neurologic symptoms and required neuroimaging were only included in the study. Neuroimaging examinations were evaluated for the presence of, infarction, hemorrhage and encephalopathy. The frequency of these findings was correlated with clinical variables, including presence of comorbidities, requirement for intensive care unit admission, and duration between admission and onset of neurologic signs and symptoms as documented in the hospital medical records.

**Results:**

A total of 135 patients underwent at least one cross-sectional imaging of the brain, the median age of these patients was 63 years, and 72% were men. Disturbed level of consciousness was the most common neurologic symptom (80.7%). Acute neuroimaging findings were found in 34 patients (25.2%) including; acute ischemic infarcts (16/135; 11.9%), intracranial hemorrhages (9/135, 6.7%), cerebral venous thrombosis (2/135; 1.5%), posterior reversible encephalopathy syndrome (1/135; 0.7%), and hypoxic-ischemic encephalopathy (6/135, 4.4%). There was no statistically significant difference in patient age (*p* = 0.062), sex (0.257), presence of comorbidities (*p* = 0.204), intensive care unit admission (*p* = 0.326) and duration between admission and onset of neurologic signs and symptoms (*p* = 0.755), in patients with positive versus negative neuroimaging studies.

**Conclusions:**

Our study showed that cerebrovascular complications, ischemic and hemorrhagic were the most frequent imaging finding in hospitalized patients with COVID-19. Knowledge about these potentially serious complications can help optimize management for these patients.

## Background

The current coronavirus-associated acute respiratory disease called coronavirus disease 2019 (COVID-19) is caused by a new virus that belongs to the species ‘severe acute respiratory syndrome-related coronaviruses (SARS-CoVs)’ and is named SARS-CoV-2 [1]. Like other respiratory viruses, it infects the epithelial cells in the upper airway, and in a good percentage of cases, it is cleared by the immune system, causing only minimal symptoms. In more vulnerable individuals, the virus affects the lower respiratory tract resulting in more severe illnesses such as pneumonia and severe respiratory distress syndrome [2].

In addition to respiratory symptoms, many neurologic manifestations of COVID-19 are increasingly reported, including headache, dizziness, confusion, seizures, and focal neurologic deficits [3–8]. Variable neuroimaging findings have been reported in patients with COVID-19 including ischemic infarct, hemorrhage, acute hemorrhagic necrotizing encephalopathy, cerebral venous thrombosis, and diffuse leukoencephalopathy with microhemorrhages [9–13].

It was found that SARS-CoV-2 shows a high affinity for the angiotensin-converting enzyme 2 (ACE2) receptor, which is expressed in the epithelium of the upper and lower airways, as well as the endothelium of the capillaries including those in the central nervous system [14]. At the same time, COVID-19 patients, especially severe cases, frequently present severe systemic manifestations such as viremia, hypoxia, cytokine storm, and coagulopathy. It is still not clear if neurologic symptoms and different neuroimaging finding result from these systemic changes or direct effects of the virus itself [15].

Our study aimed to describe variable neuroimaging findings observed in hospitalized patients with COVID-19. This may provide valuable insight into coronavirus pathogenesis and guide the early detection and treatment of neurologic diseases associated with COVID-19 infection.

## Methods

This retrospective study was approved by the institutional review board. Informed consent was waived. The study was conducted in our tertiary care hospital which is assigned to treat patients with COVID-19. We retrospectively reviewed all patients admitted between July 1 and December 30, 2020. All cases were confirmed to have COVID-19 by acquiring real-time reverse transcription-polymerase chain reaction (RT-PCR) analysis for SARS-CoV-2 of throat swab specimens. Patients presented with acute neurologic symptoms and required neuroimaging were only included in the study. Data were collected from hospital medical records include age, sex, acute neurologic symptoms, presence of comorbidities, duration between admission and onset of neurologic signs and symptoms and intensive care unit (ICU) admission.

### Imaging technique

All computed tomography (CT) examinations were obtained on a 128-section multidetector-row CT scanner (Revolution Frontier, GE Healthcare). Axial non-contrast CT studies of the head were acquired with 120-kV, automatically adjusted mAs and 0.625–1.25 mm section thickness. Imaging was obtained from the level of the skull base to the vertex. Coronal and sagittal reformats with 2-mm-thick slices were reconstructed. For CT angiography (CTA) of the head and neck; images were obtained from the level of the aortic arch through the vertex, with a slice thickness of 0.625 after injection of a dose of 80–100 mL of iodinated contrast material (320 mg/ mL) using a power injector at a rate of 4–5 mL/s. For CT venography (CTV), craniocaudal scanning was done, with a manual start when the veins are clearly visible below the skull base. Coronal and sagittal reformats with 2-mm-thick slices were reconstructed. MR imaging examinations of the brain were obtained on a 1.5 T scanner (Signa, GE Healthcare). For MR imaging; sequences performed included axial T1-weighted imaging (T1WI), axial T2 fluid-attenuation inversion recovery (FLAIR), axial T2WI, axial diffusion weighted imaging (DWI), axial susceptibility-weighted imaging (SWI), and sagittal T1WI. Gadopentetate dimeglumine (0.1 mmol/kg) was used for contrast material-enhanced studies.

### Image evaluation

All CT and MR imaging examinations obtained during the hospitalization of patients for COVID-19 were reviewed by two neuroradiologists (A.A., N.E.; 18 and 11 years of experience, respectively). Acute neuroimaging findings, including the presence of acute or subacute infarction, hemorrhage, and encephalopathy were recorded. For acute or subacute infarctions, the location and vascular territory of the infarct were recorded. For intracranial hemorrhages, the compartment of intracranial hemorrhage (parenchymal, subarachnoid, subdural or intraventricular) was defined. Posterior reversible encephalopathy syndrome (PRES) was determined by the presence of confluent T2-FLAIR hyperintensity at MRI [16]. Hypoxic-ischemic encephalopathy (HIE) was suggested at CT if there is diffuse cerebral edema with loss of normal gray-white differentiation, and at DWI when there is restricted diffusion in the basal ganglia or cerebral cortex [17]. At CTA and CTV, the location and extent of thrombosis whether arterial or venous were reported. Chronic findings, including chronic infarcts or white matter changes likely of microvascular origin based on the age of the patient, were considered incidental and not recorded.

### Data analysis

Data were described using frequency and proportion for categorical variables and using mean and range for quantitative data. Two-tailed t test was performed to evaluate significant difference in patient age for patients with positive versus negative neuroimaging findings. Chi-squared and Mann–Whitney U tests were used to measure associations between neuroimaging findings and clinical parameters including presence of previous comorbidities, intensive care unit admission and duration between admission and onset of neurologic signs and symptoms. A p value of less than 0.05 was considered statistically significant.

## Results

### Patient characteristics

A total of 135 patients met the inclusion criteria of the study, the age range was 21–88 years, the median age was 63 years, 97 were males and 38 were females. Regarding the presence of comorbidities, 95 (95/135; 70.4%) had at least one of the following chronic disorders: hypertension (73/135; 54%), diabetes (63/135; 46.7%), coronary artery disease (13/135; 9.6%) and cerebrovascular disease (2/135; 1.5%). Among 135 patients, 90 of them (66.7%) required ICU admission. The most common neurologic symptoms were disturbed level of consciousness in 109 patients (109/135; 80.7%), focal neurologic deficit in 19 patients (19/135; 14.1%), headache in 5 patients (5/135; 3.7%), visual disturbances in 4 patients (4/135; 3%), seizures in 2 patients (2/135; 1.5%), ataxia in 1 patient (1/135; 0.7%), neck stiffness in 1 patient (1/135; 0.7%), behavioral changes and agitation in 1 patient (1/135; 0.7%).

Non-contrast CT of the brain was done in 135 cases, and MRI of the brain was done in 8 patients. MRI with contrast was performed in one patient. Regarding neurovascular imaging, 7 patients had CTA of the head and neck arteries and 12 patients had CTV of the cerebral veins. Of 135 patients with neuroimaging studies; 34 patients had acute neuroimaging findings (34/135; 25.2%) and 101 patients (101/135; 74.8%) had either normal scan or chronic findings. The demographic and clinical data of all patients are illustrated in Table 1.

**Table 1:** Demographic and clinical data of patients with COVID-19 and acute neurologic manifestations who underwent neuroimaging

	Total patients underwent neuroimaging (total number = 135)	Patients without acute neuroimaging findings (total number = 101)	Patients with acute neuroimaging findings (total number = 34)
*Age range*	21–88	21–88 years	33–83 years
*Male patients*	97	70	27
*Number of patients with comorbidities*	95	74	21
Hypertension	73	63	10
Diabetes	65	55	10
Coronary disease	13	12	1
Cerebrovascular disease	2	1	1
*Neurologic manifestations*			
Disturbed level of consciousness	109	83	26
Focal neurologic deficit	19	10	9
Seizures	2	–	2
Headache	5	5	-
Visual symptoms	4	4	-
Ataxia	1	1	-
Behavioral changes	1	1	-
Neck stiffness	1	1	-
*ICU admission*	90	65	25
*Imaging examination*			
CT	135	101	34
MRI	8	5	3

Among 34 patients with acute neuroimaging findings, the age range was 33–83 years, the median age was 60 years, 27 were males and 7 were females. The most common neurologic symptoms were disturbed level of consciousness in 26 patients (26/34; 76.5%), focal neurologic deficit in 9 patients (9/34; 26.5%) and seizures in 2 patients (2/34; 5.9%). Of the 34 patients, 13 patients had no known past medical history (13/34; 38.2%) and 21 (21/34; 61.8%) had at least one of the following chronic disorders: hypertension (10/34; 29.4%), diabetes (10/34; 29.4%), coronary artery disease (1/34; 2.9%) and cerebrovascular disease (1/34; 2.9%). Out of 34 patients with acute imaging findings, 25 patients (25/ 34; 73.5%) required ICU admission for hypoxic respiratory failure, all of them were on mechanical ventilation except one patient required extracorporeal membrane oxygenation (ECMO).

### Imaging findings

Acute neuroimaging findings were detected in 34 patients (34/135; 25.2%); 16 patients (16/135; 11.9%) developed acute ischemic infarcts, nine patients (9/135, 6.7%) had intracerebral hemorrhage, two patients (2/135; 1.5%) had venous sinus thrombosis, one with posterior reversible encephalopathy syndrome (PRES) (1/135; 0.7%), and six patients (6/135, 4.4%) with hypoxic-ischemic encephalopathy pattern. Acute imaging findings are illustrated in Table 2.

**Table 2:** Neuroimaging findings of hospitalized patients with new onset of neurologic symptoms following COVID-19

Imaging findings	Number of patients (total number = 34)
*Acute ischemic infarcts*	16 (47%)
Middle cerebral artery territory	10
Anterior cerebral artery territory	3
Posterior cerebral artery territory	3
*Intracranial hemorrhage*	9 (26.5%)
Parenchymal	8
Subdural	1
Subarachnoid	2
Intraventricular	2
*Cerebral venous thrombosis*	2 (6%)
*Posterior reversible encephalopathy syndrome*	1 (2.9%)
*Hypoxic-ischemic encephalopathy*	6 (17.6%)

### Acute ischemic infarcts

**Fig. 1:**
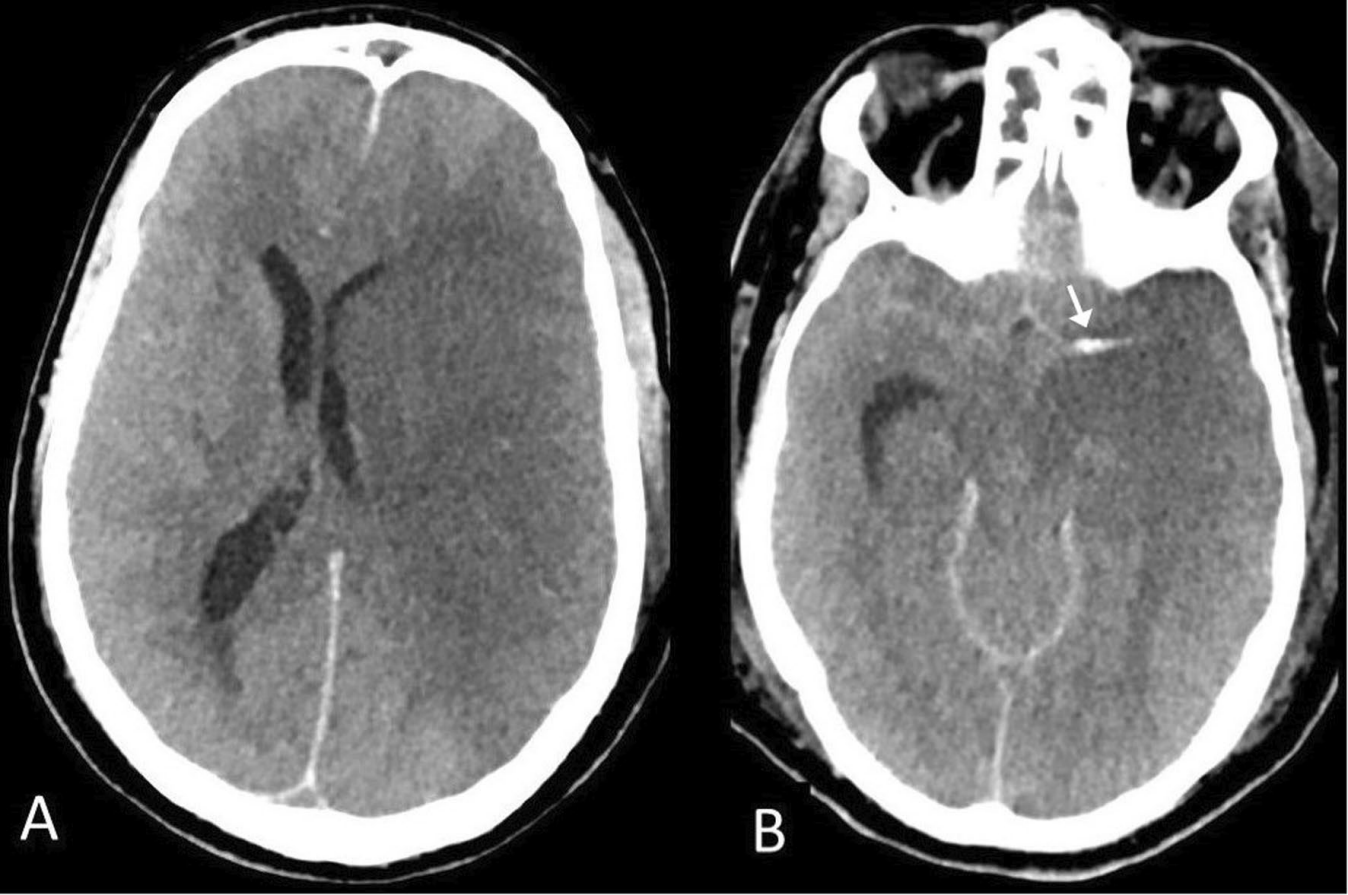
Acute ischemic infarct. 49-year-old man with COVID-19 transferred to ICU for mechanical ventilation, his neurologic condition suddenly deteriorated and the patient was found with bilaterally fixed and dilated pupils. **a** Axial CT image demonstrating large left MCA infarction. **b** Hyperdense left MCA is seen the proximal M1 segment of the left MCA (arrow), suggesting thrombus

**Fig. 2:**
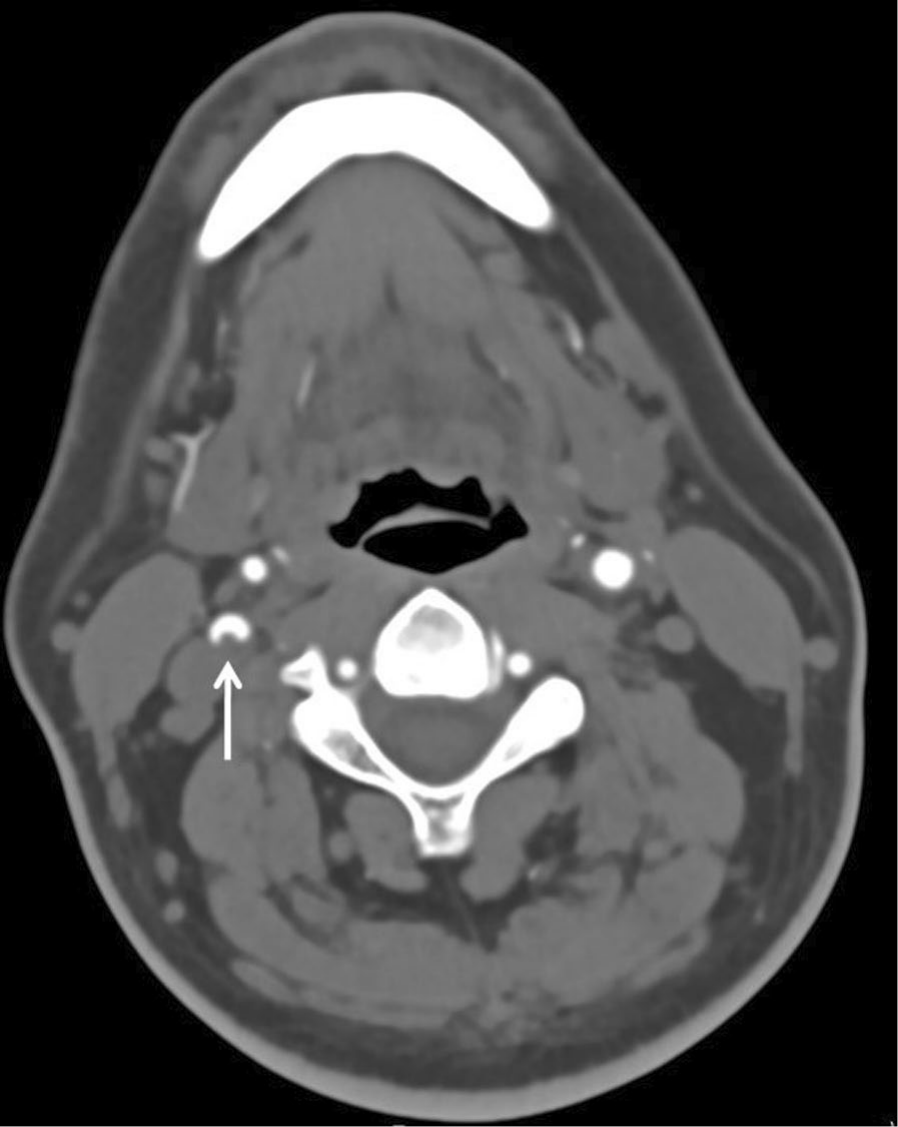
Internal carotid artery thrombus. 36-year-old-man with COVID-19 transferred to ICU on extracorporeal membrane oxygenation, developed acute onset of quadriplegia. Axial image from a CT angiogram shows acute thrombus within the right internal carotid artery (arrow). Atherosclerotic changes were not present in the remainder of the intra- and extracranial arterial vasculature

Among 16 patients with ischemic stroke, 13 infarcts involved the anterior circulation and 3 were in the posterior circulation. Large territorial infarcts (involving the whole vascular territory) were seen in 9 patients, 6 in the middle cerebral artery (MCA) territory (Fig. 1), 2 in the anterior cerebral artery (ACA) territory and one in a posterior cerebral artery (PCA) territory. CTA of the neck was done in one patient and revealed acute right internal carotid artery (ICA) thrombus without evidence of intra or extracranial atherosclerotic disease (Fig. 2). Out of these 16 patients, 4 patients were less than 50 years of age (36, 38, 43, 49 years) and had no previous comorbidities. Twelve patients had pre-existing vascular risk factors (6 had diabetes, 7 had hypertension, and 1 patient had coronary artery disease). Out of 16 patients, 11 of them required ICU admission and mechanical ventilation for hypoxic respiratory failure.

### Intracerebral hemorrhages

**Fig. 3:**
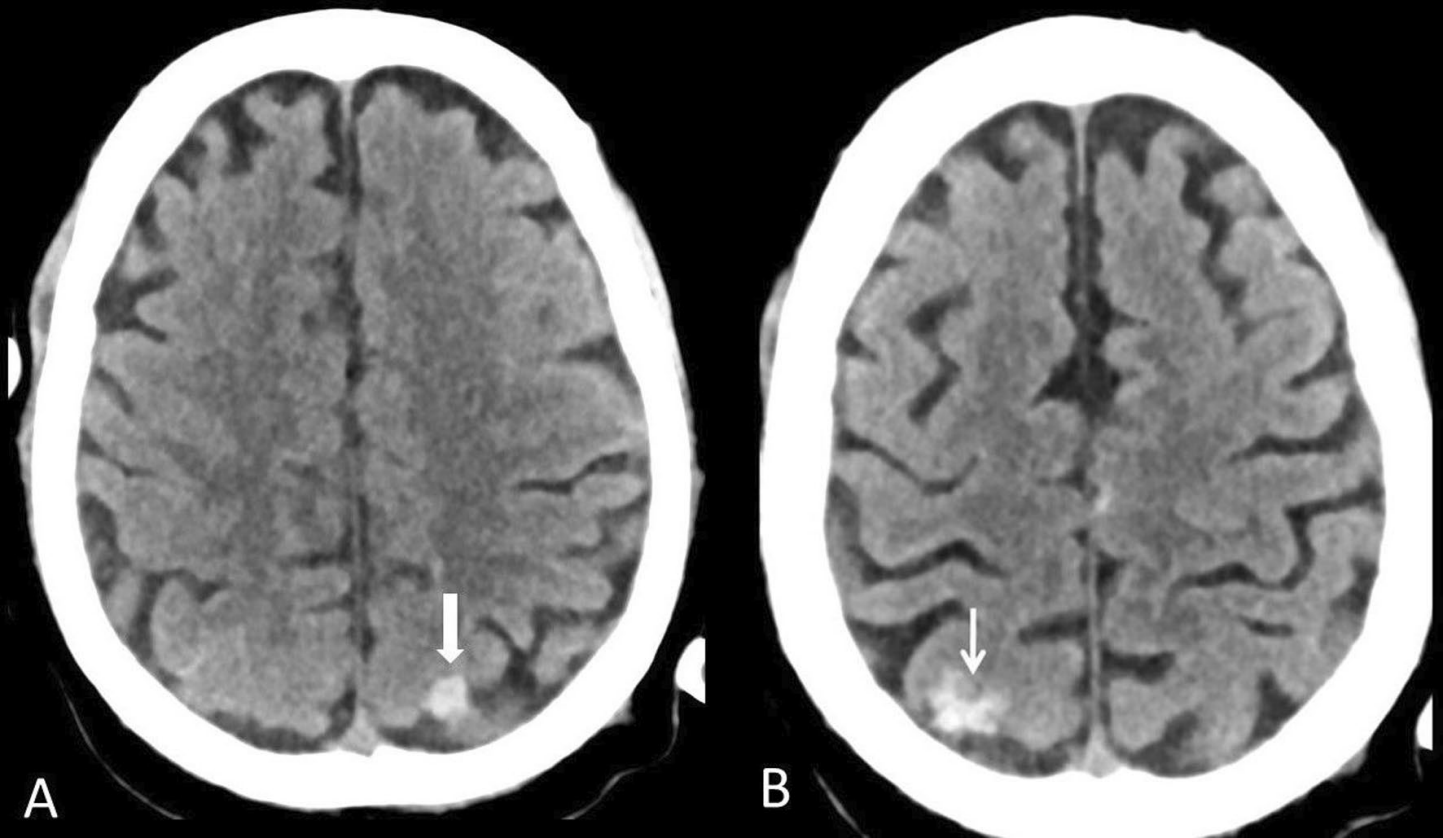
Subarachnoid hemorrhage. 64-year-old man with COVID-19 transferred to ICU and developed acute deterioration of the level of consciousness. **a, b** Axial CT brain revealed bilateral parietal isolated convexal subarachnoid hemorrhage (arrows)

**Fig. 4:**
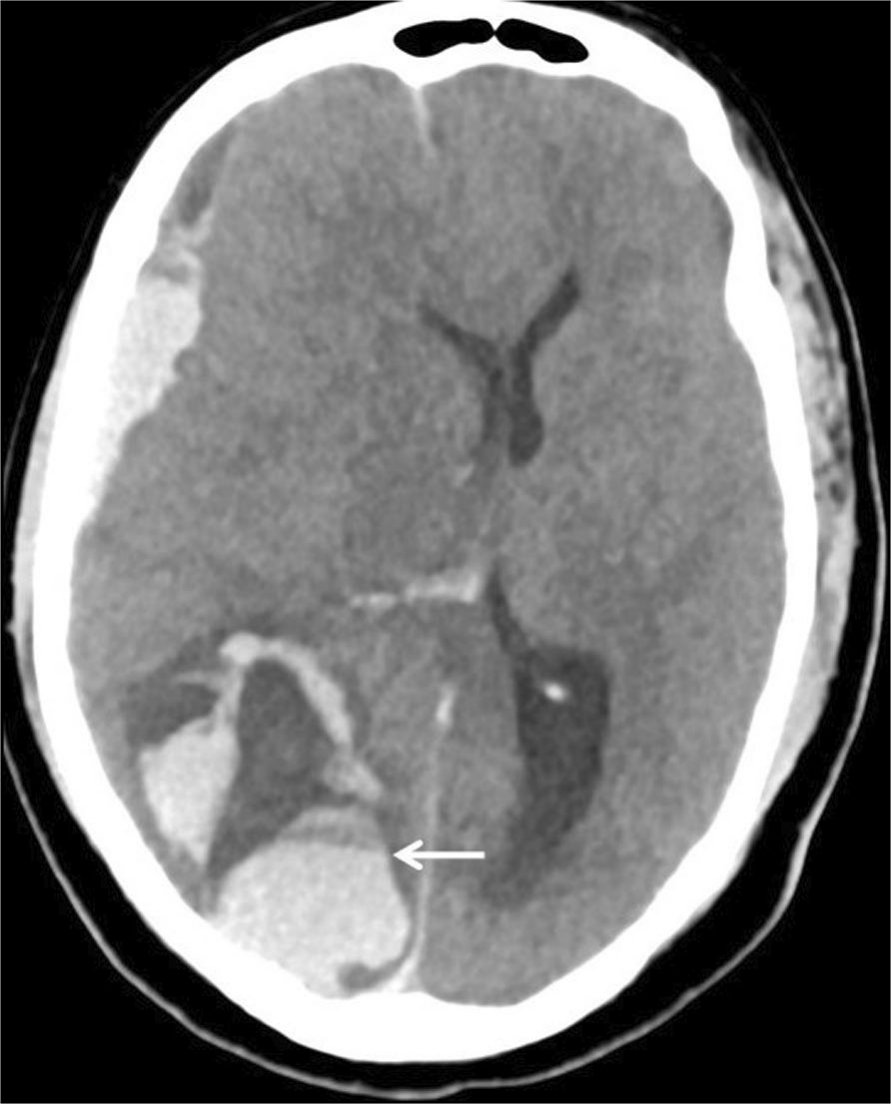
Parenchymal hemorrhage. 43-year-old man with COVID-19 transferred to ICU, developed acute myocardial infarction, and received thrombolytic therapy. He suddenly deteriorated and was found with bilaterally fixed and dilated pupils. Axial non-contrast CT of the brain revealed acute subdural hemorrhage, large occipito-parietal intraparenchymal hematoma with blood-fluid level (arrow)

Nine patients had evidence of intracranial hemorrhage on non-contrast CT of the brain. Of these, 8 patients had a parenchymal hemorrhage and one patient had isolated subarachnoid hemorrhage (SAH) (Fig. 3). Out of the 8 patients with parenchymal hemorrhages, one had associated subdural hemorrhage (SDH) and intraventricular hemorrhage (IVH) and one patient had SAH and IVH. The size of the parenchymal hematomas exceeded 5 cm in 4 patients. Two hematomas showed blood-fluid levels (Fig. 4). Three of the patients with large parenchymal hemorrhages showed evidence of diffuse hypoxic-ischemic injury. Five patients out of 9 with ICH had previous vascular risk factors (3 had diabetes and hypertension and one patient had hypertension). The remaining 4 patients had no previous comorbidities and were less than 50 years. Of the 9 patients with intracranial hemorrhage, 7 were critically ill and admitted to the ICU on mechanical ventilation, and one of them was treated with extracorporeal membrane oxygenation (ECMO). All of them were under prophylactic anticoagulation except one patient who received therapeutic anticoagulation for myocardial infarction developed during his hospital stay.

### Cerebral venous thrombosis

**Fig. 5:**
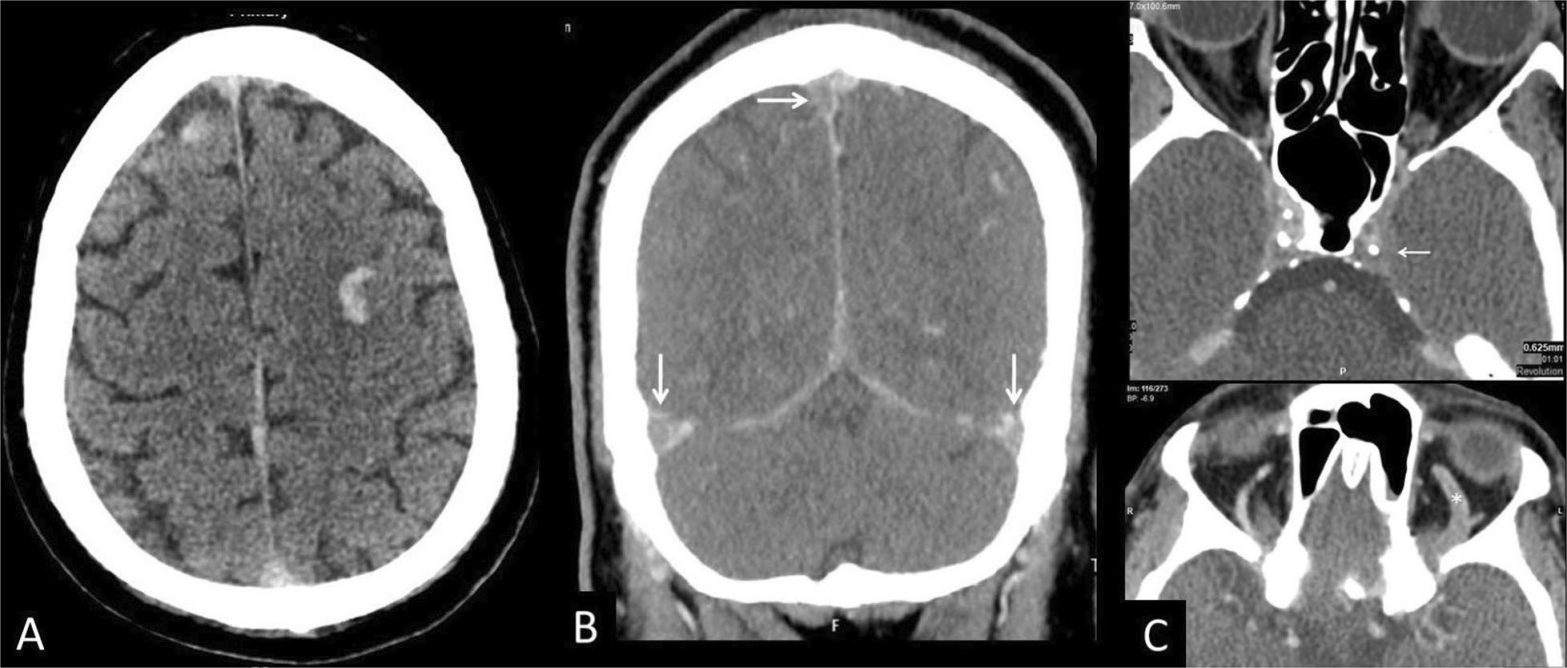
Venous sinus thrombosis. 60-year-old man presented with acute confusion. **a** Axial non-contrast CT revealed bilateral frontal hemorrhagic infarctions. **b** CTV demonstrating dural sinus thrombosis involving the superior sagittal sinus, bilateral transverse, and sigmoid sinuses (arrows). **c** The left cavernous sinus is also thrombosed (white arrow) with extension to the left superior ophthalmic vein (asterisk)

Cerebral venous sinus thrombosis was diagnosed in two patients; first case was a 60-year-old man with extensive dural sinus thrombosis involving the superior sagittal sinus, bilateral transverse, and sigmoid sinuses, as well as the left cavernous sinus extending to left superior ophthalmic vein complicated by bilateral frontal hemorrhagic venous infarction (Fig. 5); the patient was discharged around 2 weeks ago from our hospital following a short and uneventful admission for COVID-19, presented again to our emergency department with confusion. He has no other obvious risk factors for thrombosis. Second case was a 60-year-old man admitted to ICU for hypoxic respiratory failure on mechanical ventilation, CT brain was done for the sudden deterioration in the level of consciousness, and CTV revealed dural sinus thrombosis at right sigmoid sinus extending below skull base to right internal jugular vein (IJV).

### Posterior reversible encephalopathy syndrome (PRES)

**Fig. 6:**
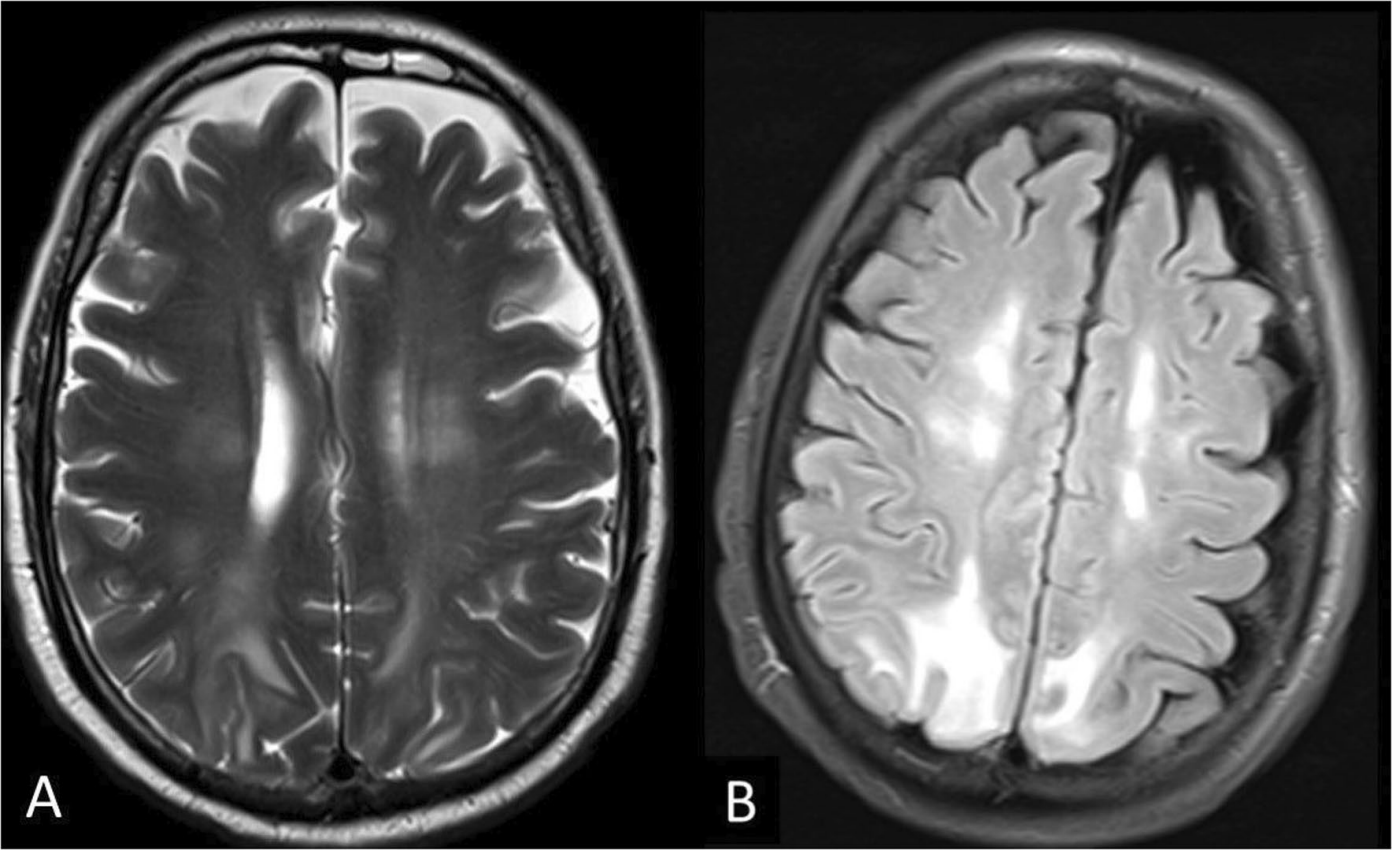
PRES. A 47-year-old man with COVID-19 admitted to ICU, and developed acute hypertension and generalized tonic–clonic convulsions for the first time. **a, b** Axial T2-weighted and FLAIR image demonstrates bilateral subcortical parieto-occipital white matter hyperintense signal extending to involve superior frontal regions

The study included one case of PRES, (47-year-old man) was admitted to the ICU and developed acute hypertension and generalized tonic colonic convulsions for the first time during his intensive care stay. Initial CT showed bilaterally Parieto-occipital hypodensity and MRI revealed parieto-occipital and superior frontal confluent T2 and FLAIR hyperintensities (Fig. 6) without diffusion restriction or blooming at SWI.

### Hypoxic-ischemic encephalopathy

**Fig. 7:**
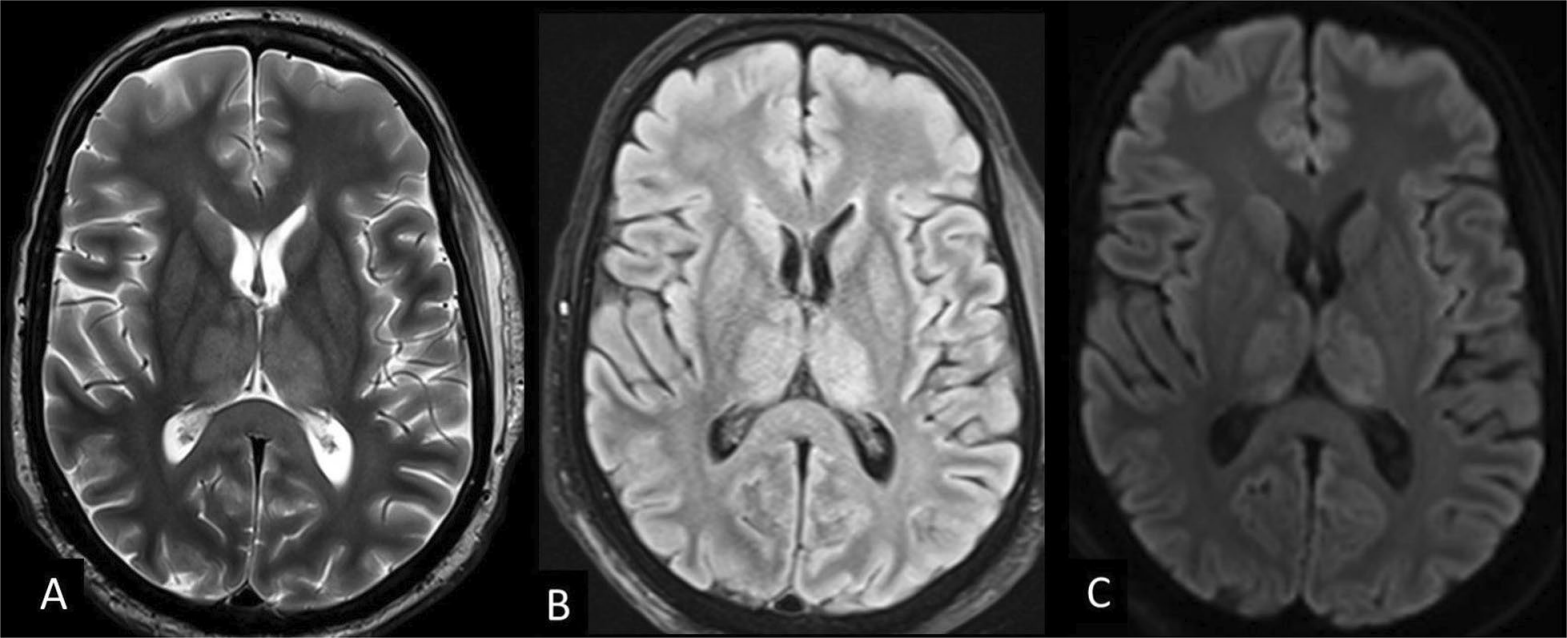
Hypoxic-ischemic encephalopathy. MRI images showing bilateral hypoxic-ischemic changes. **a, b** Axial T2-weighted and FLAIR images revealed in the basal ganglia and thalami. **c** DWI images show high diffusion signal in the basal ganglia and thalami

Hypoxic-ischemic encephalopathy was seen in 6 patients admitted to ICU with a history of cardiopulmonary arrest. CT done in 4 patients revealed diffuse brain edema with lost gray-white matter differentiation. MRI done in two patients showed signs of severe hypoxic-ischemic brain injury of the deep gray nuclei with diffusion restriction involving the basal ganglia and thalami (Fig. 7).

### Correlation analysis

There was no statistically significant difference in patients’ age (*p* = 0.062), sex (*p* = 0.257), presence of comorbidities (*p* = 0.204), ICU admission (*p* = 0.326) and duration between admission and onset of neurologic signs and symptoms (*p* = 0.755) in patients with positive versus negative neuroimaging studies (Table 3).

**Table 3:** Comparison between patients with coronavirus disease 2019 with and without acute neuroimaging findings

	Acute neuroimaging findings	No acute neuroimaging findings	Statistical significance
Number of patients (%)	34 (25.2%)	101 (74.8%)	–
Mean age ± SD	58 ± 13.4	63.1 ± 13.9	0.062
Gender, n (%)	27 males (79.4%)	70 males (69.3%)	0.257
Presence of comorbidities, n (%)	21 (61.8%)	74 (73.3%)	0.204
Intensive care unit admission, n (%)	25 (73.5%)	65 (64.4%)	0.326
Duration between admission and onset of neurologic signs and symptoms, mean (days) ± SD	19 ± 15.9	15.9 ± 10.5	0.755

*SD* standard deviation

## Discussion

The mechanism of neurologic complications in patients with COVID-19 is complex and multifactorial. As the COVID-19 pandemic continues to progress, cerebrovascular manifestations of COVID-19 became more evident. Although COVID-19 has been observed to affect the respiratory system primarily, neurologic involvement has already been reported [18]. In the present study, a number of patterns in the brain CT and MRI were seen. On CT, patterns consisted of acute/subacute infarcts, parenchymal, subdural, and subarachnoid hemorrhage. On CTA and CTV, carotid artery thrombus and venous sinus thrombosis were seen. On MRI, hypoxic-ischemic changes and PRES changes were detected.

A total of 135 patients developed acute neurologic manifestation and underwent neuroimaging with either CT, MR imaging, or both, 25.2% of them had acute imaging findings. Ischemic stroke (11.9%) was the most common acute finding. Several hypotheses suggested that the pathogenesis of stroke in COVID-19 may be attributed to the development of coagulopathy or endothelial dysfunction [9, 14]. Systemic inflammation and cytokine storm triggers the coagulation system with subsequent development of a hypercoagulable state. In addition, this inflammatory state can damage the capillary endothelium resulting in dysregulation of its anti-thrombotic properties. Both coagulopathy and endothelial dysfunction account for the increased risk of thrombotic disease involving either the arterial or venous circulations in patients with COVID-19. Moreover, patients with COVID-19 hospitalized in intensive care units are at higher risk of venous and arterial thrombotic events despite prophylactic anticoagulation [15, 19].

Cerebrovascular events (both ischemic and hemorrhagic) were found to be the most common neuroradiologic abnormality seen among COVID-19 patients [20]. In our study, 11.9% patients developed acute ischemic infarcts. Chougar et al. [21] reported acute ischemic infarcts in 7.6% of patients with acute neurologic manifestations. In a study of 108 hospitalized COVID-19 patients who underwent neuroimaging, 31% demonstrated acute ischemia [22]. As in other series [23], most of our patients with ischemic infarcts (12/16) had cerebrovascular comorbidities, like hypertension and diabetes. However, growing evidence suggests that COVID-19 is associated with acute stroke, even in patients at low risk for stroke. Four out of 16 patients with ischemic stroke in our study were less than 50 years of age and had no previous comorbidities. Oxley et al. [24] reported five cases of stroke in patients younger than 50 who had no previous medical history.

Large territorial infarcts were seen in 9 patients in the current study. Kihira et al. [25] found that patients with COVID-19 had large vessel occlusion stroke risk 2.4 times that of patients without COVID-19 after control for race and ethnicity in his multivariate analysis. Our experience supports other observations of patients with COVID-19 developing acute stroke secondary to acute thrombus within the internal carotid artery. Gulko et al. [26] illustrated two cases with imaging findings of acute thrombus at the proximal internal carotid arteries without other signs of intra- or extracranial atherosclerotic disease similar to our case.

In addition to arterial thrombosis, several studies have observed high rates of venous thromboembolism in critically ill patients with COVID-19 who otherwise lack the classic risk factors for venous thromboembolism [19, 27]. In our study, two patients developed cerebral venous thrombosis (CVT), one of them was admitted to the ICU on mechanical ventilation for hypoxic respiratory failure. Both patients had dural sinus thrombosis. In general, the most common predisposing factor of dural sinus thrombosis is hypercoagulability in the context of a prothrombotic condition which is, in our cases, possibly induced by SARS-COV2 infection. Early identification is critical because prompt anticoagulation can improve outcomes, as it is a potentially reversible condition. As CVT is one of the few causes of cerebral hemorrhage that call for anticoagulation therapy, diagnostic certainty is important so that appropriate treatment can be started. Diagnosis is difficult because the clinical manifestations of CVT are nonspecific, such as headache, seizures, decreased level of consciousness, and focal neurologic deficits. For this reason, imaging is crucial to diagnosis, and radiologists must be able to identify the findings that raise suspicion of CVT (anticoagulation therapy) early to avoid complications and even death [28, 29].

Intracranial hemorrhage (ICH) reported in patients with COVID-19 is a devastating event. Therapeutic anticoagulation is usually initiated to treat thrombotic complications such as deep vein thrombosis, pulmonary embolism, stroke, and acute myocardial infarction [30]. Patients with COVID-19 who develop the severe disease may have a series of complications such as renal failure or liver dysfunction, which can affect both venous thromboembolism and bleeding status. Regular monitoring and adjustment of thromboprophylaxis is crucial to prevent complications [30]. In the current study, 6.7% of patients with acute neurologic symptoms had intracerebral hemorrhage. In one study [31], 4.4% of patients with neuroimaging had ICH, the majority of them received therapeutic anticoagulation. Radmanesh et al. [32] reported acute intracranial hemorrhage in 4.5% of patients with acute neurologic symptoms. ICH secondary to anticoagulation therapy is an important iatrogenic complication. The blood-fluid level was seen in two patients in our series. This is caused by the dependent separation of heavier blood from a lighter serous fluid. This neuroimaging finding is characteristic for patients who have been treated with anticoagulants [33]. In our cases, one patient received therapeutic anticoagulation for acute myocardial infarction, a complication that issued during his hospital stay and the other patient had prophylactic anticoagulation. We reported only one case of isolated convexity SAH at high parietal regions bilaterally. Nawabi et al. [34] found isolated cortical SAH to be the most frequent hemorrhage type in their study most of them localized along the convexity.

PRES was suggested diagnosis in one case with acute hypertension and seizures during his ICU stay. Patients with PRES can present clinically by headache, visual disturbances, disturbed level of consciousness, or seizures. At MRI, it appears as edema involving the cortex and subcortical white matter commonly at the parieto-occipital lobes but may be atypically seen at the frontal and temporal lobes, brainstem or cerebellum. It usually appears bilateral and asymmetric, without diffusion restriction [35]. In patients with COVID-19, PRES can be explained in relation to multiple risk factors. The cytokine storm of COVID-19 causes endothelial dysfunction leading to increased permeability of the blood–brain barrier (BBB) rendering susceptible to blood pressure changes. Renal failure is a common complication associated with critically ill patients with COVID-19 could be another explanation [36–38].

In a study by Yoon et al. [39], the prevalence of ICU admission and intubation was significantly higher for patients with acute neuroimaging findings than for patients without acute neuroimaging findings. In the current study, number of patients requiring ICU admission was higher among those with acute neuroimaging finding (73.5%) than patients without acute neuroimaging findings (64.4%), however, this difference was not statistically significant (*p* = 0.326).

This study has some limitations, first, the main imaging modality in our study was non-contrast CT of the brain. Only a few cases underwent MRI. MRI is known to have better sensitivity than CT but similar to other policies worldwide, MR imaging use was limited to patients with COVID-19 with the highest clinical urgency. Second, CSF analysis was not done in our patients. This procedure was limited during the pandemic to avoid the risk of cross-infection from invasive procedure and the risk of complications including bleeding as many patients are on anticoagulant therapy. Third, this study had inherent limitations related to its retrospective nature. Further prospective studies are recommended.

## Conclusions

As the novel coronavirus pandemic spreads, neurologic complications are increasingly recognized, and understanding the exact underlying mechanism is still challenging. Of our patients with COVID-19 undergoing neuroimaging, we observed ischemic strokes and intracranial hemorrhages were the most frequent imaging findings. Familiarity with these potentially serious complications can help optimize the management of patients with COVID-19 with neurologic manifestations. Future prospective studies would help to establish treatment guidelines in these critical cases.

## Data Availability

The datasets used and/or analyzed during the current study are available from the corresponding author on reasonable request.

## Abbreviations

COVID 19: Coronavirus disease 2019
SARS-CoV: Severe acute respiratory syndrome-related coronaviruse
ACE2: Angiotensin-converting enzyme 2
RT-PCR: Real-time reverse transcription-polymerase chain reaction
ICU: Intensive care unit
CT: Computed tomography
CTV: CT venography
CTA: CT angiography
MRI: Magnetic resonance imaging
T1WI: T1-weighted imaging
FLAIR: Fluid-attenuation inversion recovery
T2WI: T1-weighted imaging
DWI: Diffusion weighted imaging
SWI: Susceptibility-weighted imaging
PRES: Posterior reversible encephalopathy syndrome
HIE: Hypoxic-ischemic encephalopathy
ECMO: Extracorporeal membrane oxygenation
MCA: Middle cerebral artery
ACA: Anterior cerebral artery
PCA: Posterior cerebral artery
ICA: Internal carotid artery
SAH: Subarachnoid hemorrhage
SDH: Subdural hemorrhage
IVH: Intraventricular hemorrhage
IJV: Internal jugular vein
CVT: Cerebral venous thrombosis
ICH: Intracranial hemorrhage
